# Supplementary material for: Transition Mutations in the hTERT Promoter Are Unrelated to Potential i-motif Formation in the C-Rich Strand
Source: Biomolecules. 2023 Aug 25;13(9):1308. doi: 10.3390/biom13091308 (PMC10526324; doi:10.3390/biom13091308)
Supplement: Supplementary file 1 [file biomolecules-13-01308-s001.zip › biomolecules-2514096-supplementary.pdf]

biomolecules

MDPI

Article

Transition Mutations in the hTERT Promoter Are Unrelated to Potential i-motif Formation in the C-Rich Strand

James W. Conrad [1,†], Mark L. Sowers [1,2,†], Dianne Y. Yap [1], Ellie Cherryhomes [1], B. Montgomery Pettitt [1,3], Kamil Khanipov [1] and Lawrence C. Sowers [1,4,*]

[1] Department of Pharmacology and Toxicology, The University of Texas Medical Branch, Galveston, TX 77555, USA; jamconra@utmb.edu (J.W.C.); mlsowers@utmb.edu (M.L.S.); dyyap@utmb.edu (D.Y.Y.); eccherry@utmb.edu (E.C.); mpettitt@utmb.edu (B.M.P.); kakhanip@utmb.edu (K.K.)
[2] MD-PhD Combined Degree Program, The University of Texas Medical Branch, Galveston, TX 77555, USA
[3] Department of Biochemistry and Molecular Biology, The University of Texas Medical Branch, Galveston, TX 77555, USA
[4] Department of Internal Medicine, The University of Texas Medical Branch, Galveston, TX 77555, USA
* Correspondence: lasowers@utmb.edu
† These authors contributed equally to this work.

Table of Contents	Pg
Table S1. Oligonucleotides used in this study	2
Table S2. Rate constants for single-stranded and double-stranded DNA	3
Figure S1. pH titration of partial length hTERT sequences	4
Figure S2. CD spectra of partial length hTERT sequences	5
Table S3. Synthesis of hTERT oligonucleotides	6
Table S4. Dunn's multiple comparisons test	7
Table S5. Maldi-MS of hTERT oligonucleotides	8
Associated Maldi-MS spectra	9-13

Table S1. Oligonucleotides used in this study.

**Name**	**Purpose**	**Sequence (5’-3’)**
1 (I)	hTERT-21mer-3'T	CCCCGCCCCGTCCCGACCCCT
2 (II)	hTERT-21mer-3'G	GCCCCGTCCCGACCCCTCCCG
3 (III)	hTERT-23mer-3'G	TCCCGACCCCTCCCGGGTCCCCG
4 (IV)	hTERT-23mer-3'A	ACCCCTCCCGGGTCCCCGGCCCA
5 (V)	hTERT-25mer-3'T	TCCCGGGTCCCCGGCCCAGCCCCCT
6 (VI)	hTERT-27mer-3'T	TCCCCGGCCCAGCCCCCTCCGGGCCCT
7 (VII)	hTERT-25mer-3'A	GCCCAGCCCCCTCCGGGCCCTCCCA
8 (VIII)	hTERT-26mer-3'T	GCCCCCTCCGGGCCCTCCCAGCCCCT
9 (IX)	hTERT-19mer-3'C	GCCCTCCCAGCCCCTCCCC
Seq-hTERT-C-rich	Sequencing oligo	ATGTGTAACAG-TTCCTGCCATACTGGTCCCCGCCCCGTCCCGACCCCTCCCGGGTCCCCGGCCCAGCCCCCTCCGGGCCCTCCCAGCCCCTCCCCGACGTCCACCTGGAA-GACTCCAGGTCAG
Seq-hTERT-G-rich	Sequencing oligo	CTGACCTGGAGTCTTCCAGGTGGACGTCGGGGAGGGGCTGG-GAGGGCCCGGAGGGGGCTGGGCCGGGGACCCGGGAGGGGTCGGGAC-GGGGCGGGGACCAGTATGGCAGGAACTGTTACACAT
Primer –F5	Step 1 PCR primer	TCGTCGGCAGCGTCAGATGTGTATAAGAGACAGCAACTACATGTG-TAACAGTTCCTGC
Primer –R2	Step 1 PCR primer	GTCTCGTGGGCTCGGAGATGTGTATAAGAGACAGCTCCTGACCTGGAG-TCTTCCAG
Index Primer -F	Step 2 index PCR	AATGATACGGCGACCACCGAGATCTACACXXXXXXXXTCGTCGG-CAGCGTC
Index Primer -R	Step 2 index PCR	CAAGCAGAAGACGGCATACGAGATXXXXXXXXGTCTCGTGGGCTCGG

Table S2. Rate constants for single-stranded and double-stranded DNA incubated at 37°C for 11 days at three different pH. The average rate constants across all 46 cytosines is given and the rate constants for the mutational hotspots C250 and C228.

pH	AVG k (x 10^{-4})	SD (x 10^{-4})	% DEV	C250 Cytosine 22	C228 Cytosine 44	Reads (#)
ssDNA at 37 °C for 11 d						
7	4.94	2.13	43.3	6.83	4.12	185,169
6	3.65	1.57	42.6	3.70	4.99	225,497
5	8.31	3.38	40.6	11.5	8.22	170,283
dsDNA at 37 °C for 11 d						
7	3.81	2.62	68.7	6.90	5.60	118,533
6	3.83	2.50	65.0	8.02	2.37	139,337
5	6.74	2.24	33.0	9.81	6.36	142,896

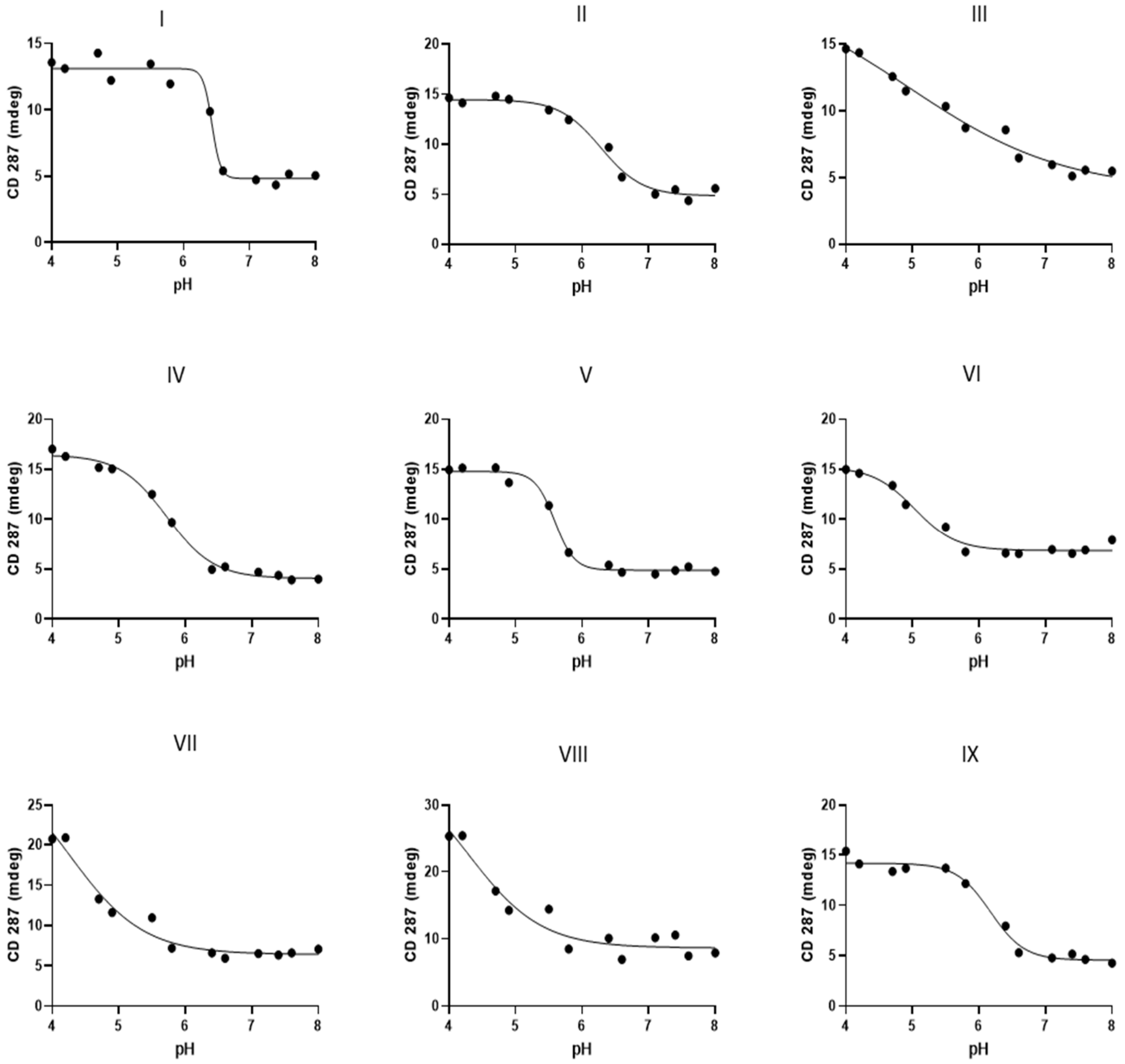

Figure S1. pH titration of partial length hTERT sequences to determine the i-motif pKa.

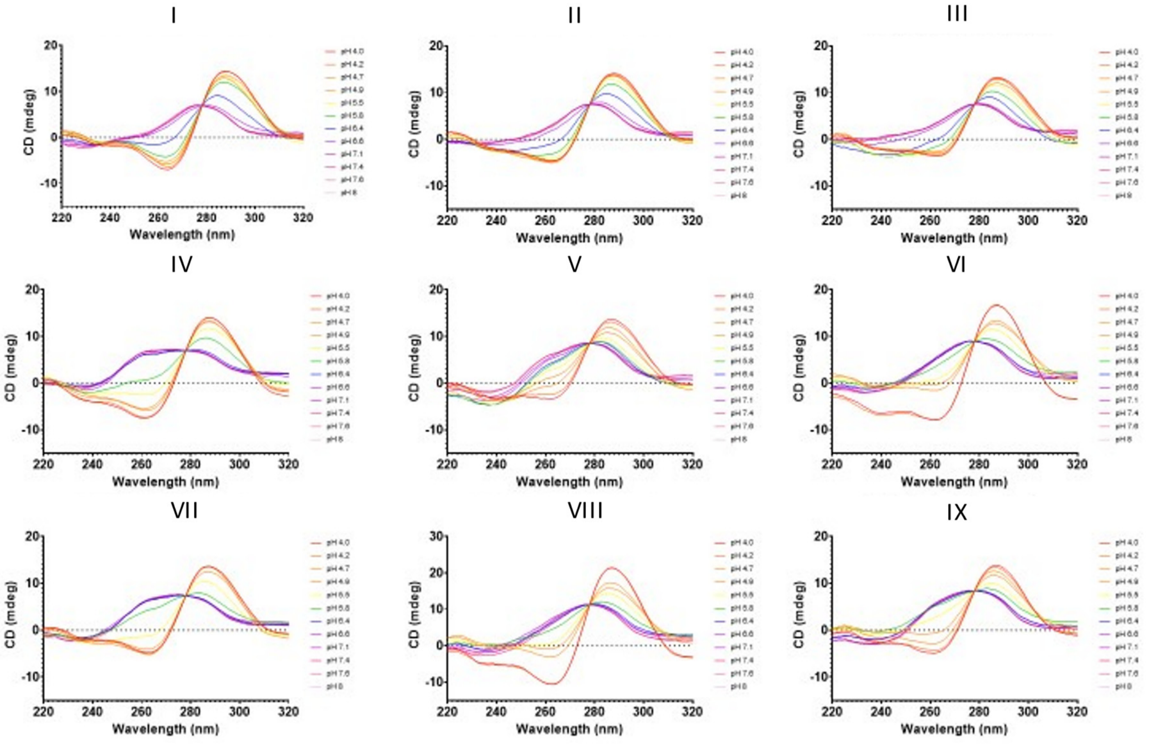

Figure S2. CD spectra of partial length hTERT sequences at pH 4-8.

Table S3. Synthesis of hTERT oligonucleotides

	Oligonucleotide	Sequence	Synthesis Conditions
1	hTERT-21mer-3’T	5'-CCCCGCCCCGTCCCGACCCCT-3'	Standard B
2	hTERT-21mer-3'G	5'-GCCCCGTCCCGACCCCTCCCG-3'	Standard B
3	hTERT-23mer-3'G	5'-TCCCGACCCCTCCCGGGTCCCCG-3'	Standard A
4	hTERT-23mer-3'A	5'-ACCCCTCCCGGGTCCCCGGCCCA-3'	Standard A
5	hTERT-25mer-3'T	5'-TCCCGGGTCCCCGGCCCAGCCCCCT-3'	Standard B
6	hTERT-27mer-3'T	5'-TCCCCGGCCCAGCCCCCTCCGGGCCCT-3'	Standard B
7	hTERT-25mer-3'A	5'-GCCCAGCCCCCTCCGGGCCCTCCCA-3'	Standard A
8	hTERT-26mer-3'T	5'-GCCCCCTCCGGGCCCTCCCAGCCCCT-3'	Standard A
9	hTERT-19mer-3'C	5'-GCCCTCCCAGCCCCTCCCC-3'	Standard A

All oligonucleotides were synthesized on an Expedite 8909 DNA synthesizer.

Standard A: Oligonucleotides were synthesized using standard phosphoramidites (Bz-dA, Bz-dC, iBu-dG, dT) and deprotected in ammonium hydroxide at 55°C for 16 hours.

Standard B: Oligonucleotides were synthesized using standard phosphoramidites (Bz-dA, Bz-dC, iBu-dG, dT) and deprotected in ammonium hydroxide at room temperature for 65 hours.

Purification and detritylation by C18 Sep-pak: C18 Sep-pak cartridge (Waters WAT020515) was prepared by washing with acetonitrile (5 mL) and 1 M triethylammonium acetate (10 mL). The crude DMT-on oligonucleotide was loaded onto the cartridge in 1 M triethylammonium acetate (2 mL) then failure sequences were eluted with 10% ammonium hydroxide (5 mL) and water (5 mL). Detritylation was done with 2% trifluoroacetic acid (5 mL) and the cartridge washed with water (5 mL). The DMT-off oligonucleotide was then eluted with acetonitrile in water (2 mL of 20% ACN, 4 mL of 50% ACN).

Table S4. Dunn's multiple comparisons test of C>T transition rates between treatments.

Dunn's multiple comparisons test	Adjusted P Value
ssDNA pH 7 95°C vs. ssDNA pH 5 37 °C	<0.0001
ssDNA pH 7 95°C vs. ssDNA pH 6 37 °C	<0.0001
ssDNA pH 7 95°C vs. ssDNA pH 7 37 °C	<0.0001
ssDNA pH 7 95°C vs. dsDNA pH 5 37 °C	<0.0001
ssDNA pH 7 95°C vs. dsDNA pH 6 37 °C	<0.0001
ssDNA pH 7 95°C vs. dsDNA pH 7 37 °C	<0.0001
ssDNA pH 5 37 °C vs. ssDNA pH 6 37 °C	<0.0001
ssDNA pH 5 37 °C vs. ssDNA pH 7 37 °C	0.0036
ssDNA pH 5 37 °C vs. dsDNA pH 5 37 °C	>0.9999
ssDNA pH 5 37 °C vs. dsDNA pH 6 37 °C	<0.0001
ssDNA pH 5 37 °C vs. dsDNA pH 7 37 °C	<0.0001
ssDNA pH 6 37 °C vs. ssDNA pH 7 37 °C	0.8593
ssDNA pH 6 37 °C vs. dsDNA pH 5 37 °C	<0.0001
ssDNA pH 6 37 °C vs. dsDNA pH 6 37 °C	>0.9999
ssDNA pH 6 37 °C vs. dsDNA pH 7 37 °C	>0.9999
ssDNA pH 7 37 °C vs. dsDNA pH 5 37 °C	0.1793
ssDNA pH 7 37 °C vs. dsDNA pH 6 37 °C	>0.9999
ssDNA pH 7 37 °C vs. dsDNA pH 7 37 °C	>0.9999
dsDNA pH 5 37 °C vs. dsDNA pH 6 37 °C	0.0001
dsDNA pH 5 37 °C vs. dsDNA pH 7 37 °C	0.0004
dsDNA pH 6 37 °C vs. dsDNA pH 7 37 °C	>0.9999

Table S5. Maldi-MS of hTERT oligonucleotides

Oligonucleotide	Expected (M+H)	Observed (M+H)	Δ
hTERT-21mer-3’T	6185.81	6184.54	-1.27
hTERT-21mer-3'G	6225.83	6223.03	-2.8
hTERT-23mer-3'G	6859.21	6857.06	-2.15
hTERT-23mer-3'A	6868.22	6863.15	-5.07
hTERT-25mer-3'T	7477.58	7476.58	-1
hTERT-27mer-3'T	8055.93	8053.6	-2.33
hTERT-25mer-3'A	7446.57	7443.07	-3.5
hTERT-26mer-3'T	7726.73	7720.83	-5.9
hTERT-19mer-3'C	5567.43	5564.07	-3.36

Desalting of C18 Sep-pak purified oligonucleotides: Micro BioSpin P6 column (BioRad 732-6221) was prepared by centrifugation at 1000G for 2 minutes. Water (500 µL) was added and the column washed by centrifugation at 1000G for 1 minute then a second wash was done. 1 OD of purified oligonucleotide in water (100 µL) was loaded and eluted into a new collection tube by centrifugation at 1000G for 4 minutes. The water was evaporated under reduced pressure and the oligo resuspended in water (25 µL).

Maldi Sample Preparation: 0.4 OD of HPLC purified or P6 BioSpin column desalted oligo in water (10 µL) was mixed with desalting ion exchange resin (2 µL) for 1 hour.

Desalting Ion Exchange Resin: Prepare slurry of cation exchange resin (6 g) in 50 / 50 acetonitrile / water (5 mL), pour into column and let settle (gravity). Wash resin with 50 / 50 acetonitrile / water (3-4 column volumes), 5% ammonium hydroxide (2 x 10 mL), 2 M ammonium acetate (3 x 10 mL) and water (3-4 column volumes). Aliquot into 1 mL fractions (50% suspension) and store -20°C.

Matrix: 3-hydroxypicolinic acid (70 mg) and ammonium citrate (10 mg) in 50/50 acetonitrile / water (1 mL) with 0.1% trifluoroacetic acid

The Maldi plate was spotted with HPA matrix (1 µL) and allowed to dry. The sample (1µL) was spotted on top of the matrix and allowed to dry before running on Bruker Autoflex Maldi-MS in positive mode.

Expected (M+H) values were calculated using average molecular weights.

MALDI-MS+ spectra of oligonucleotides

hTERT-21mer-3’T

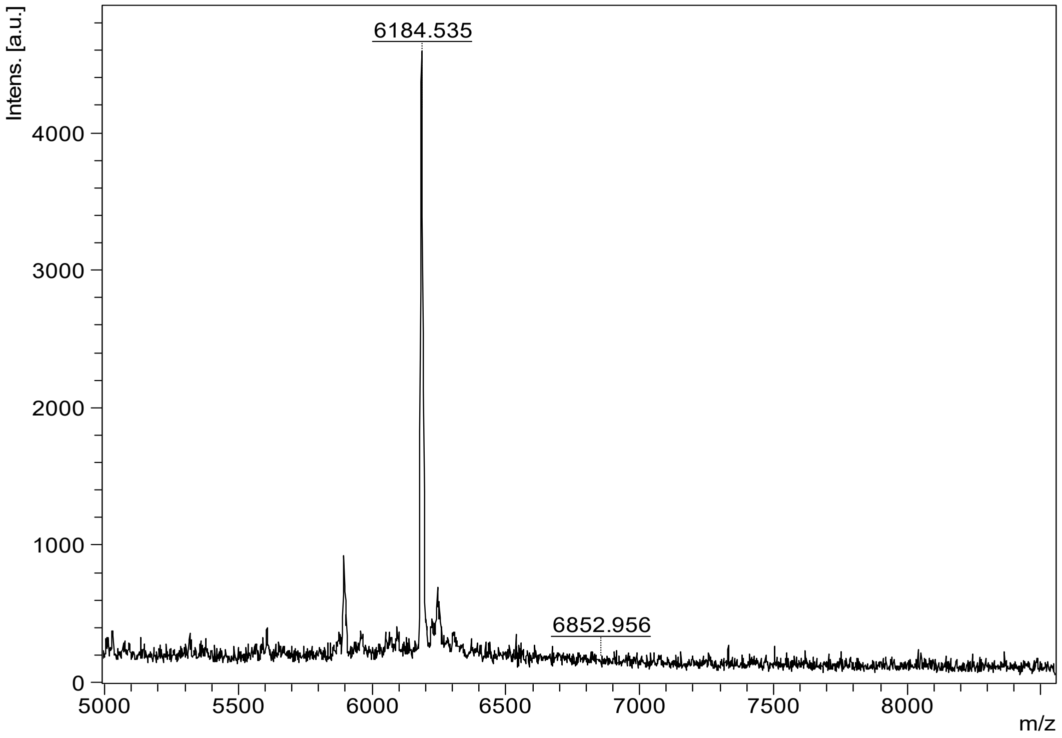

hTERT-21mer-3'G

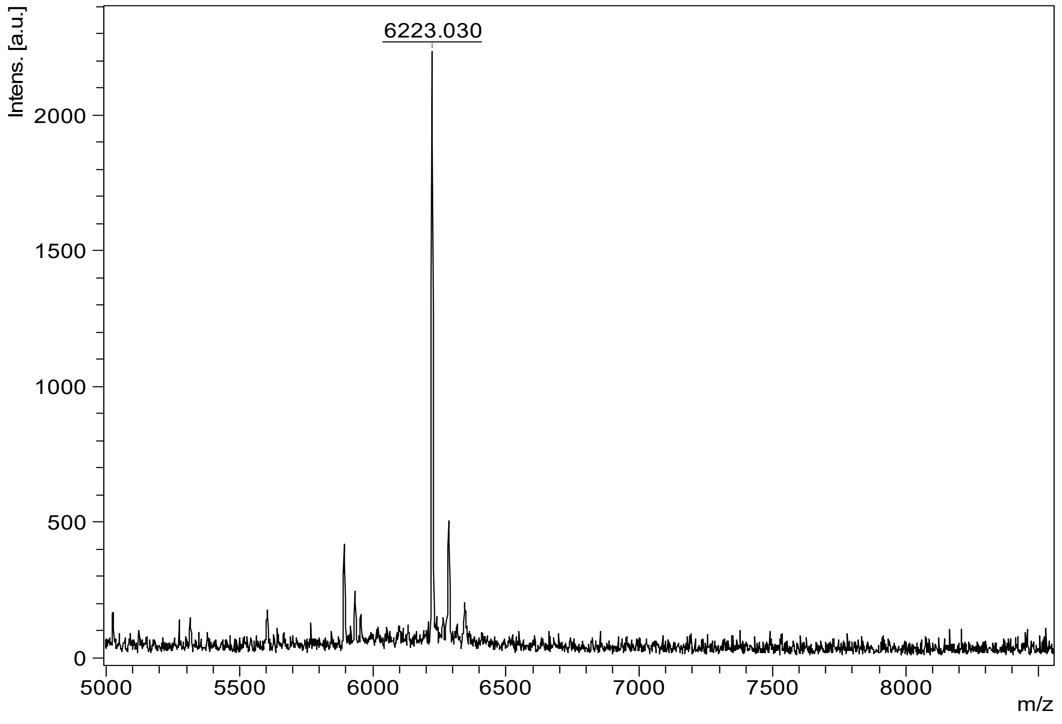

hTERT-23mer-3'G

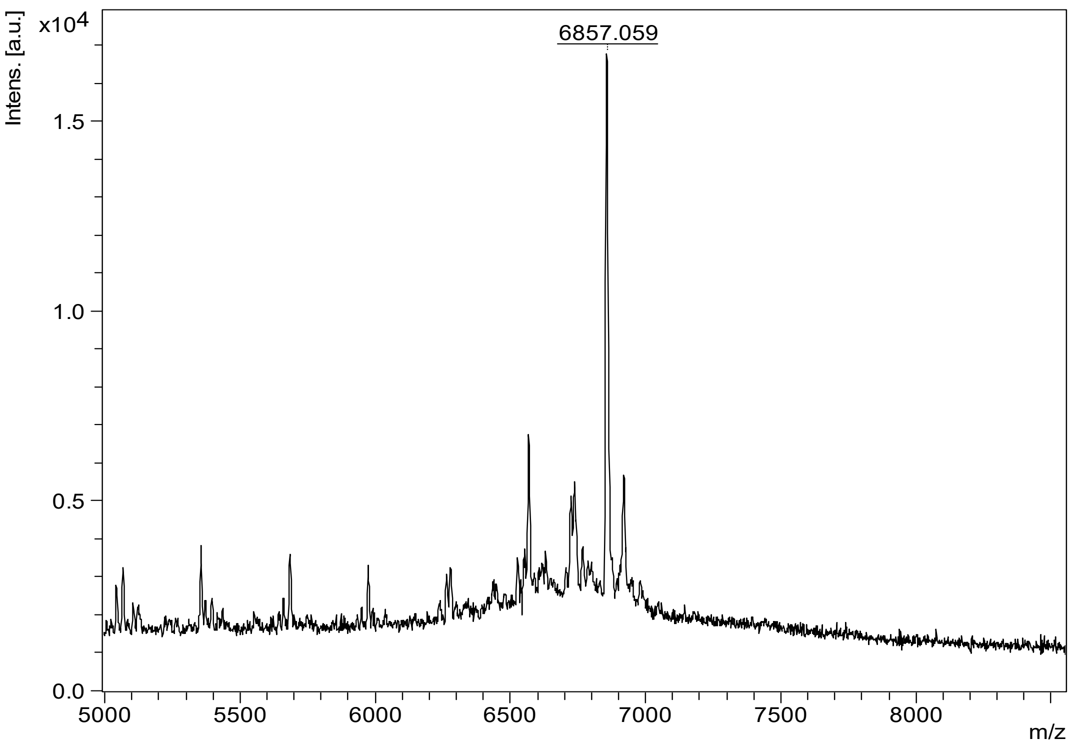

hTERT-23mer-3'A

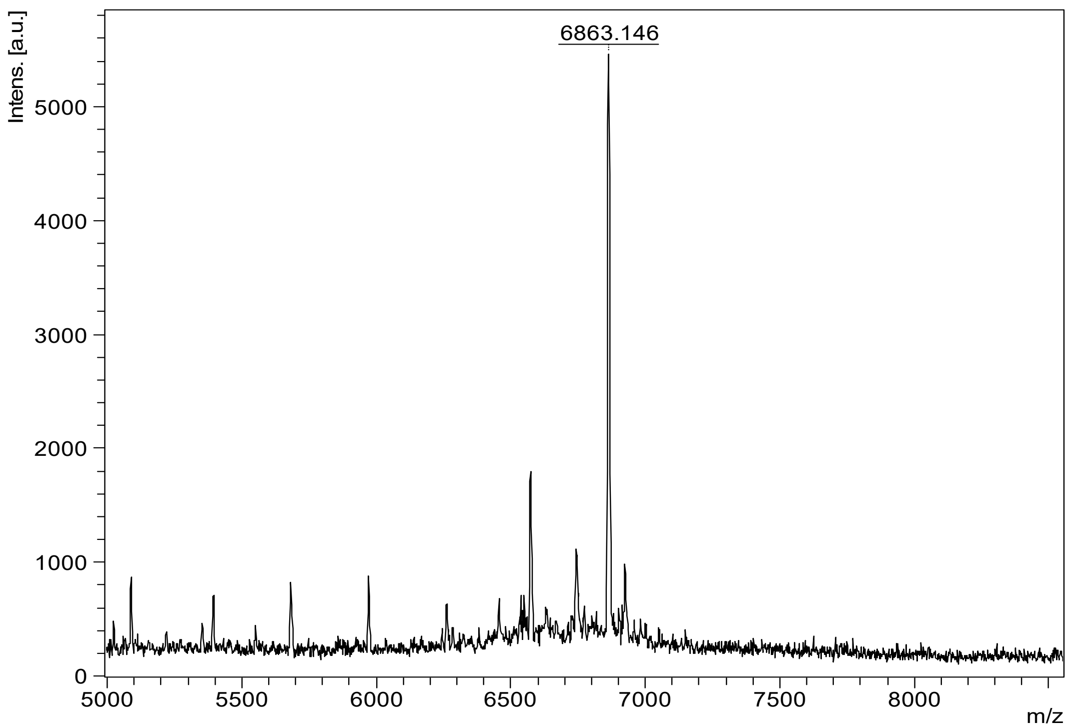

hTERT-25mer-3'T

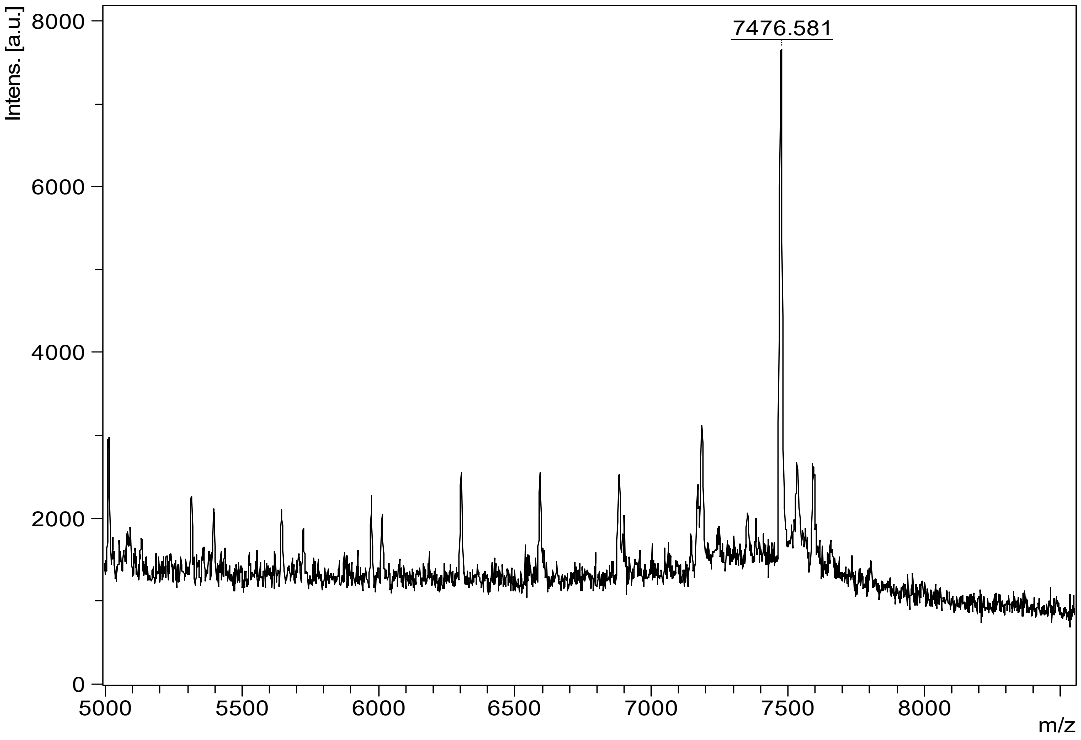

hTERT-27mer-3'T

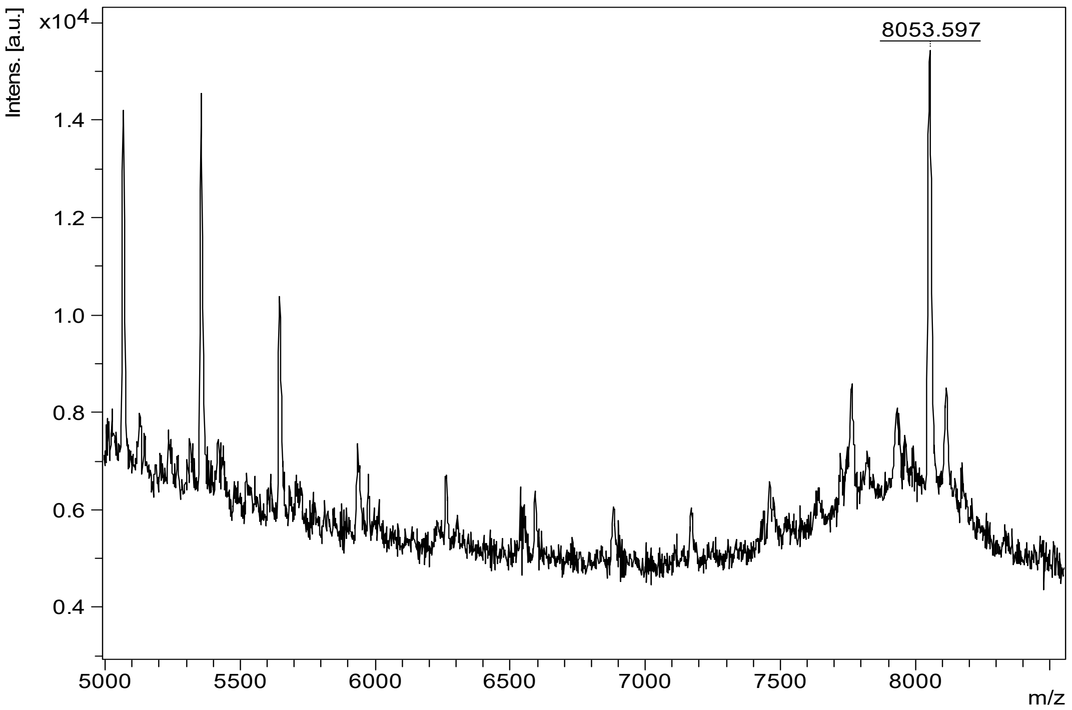

hTERT-25mer-3'A

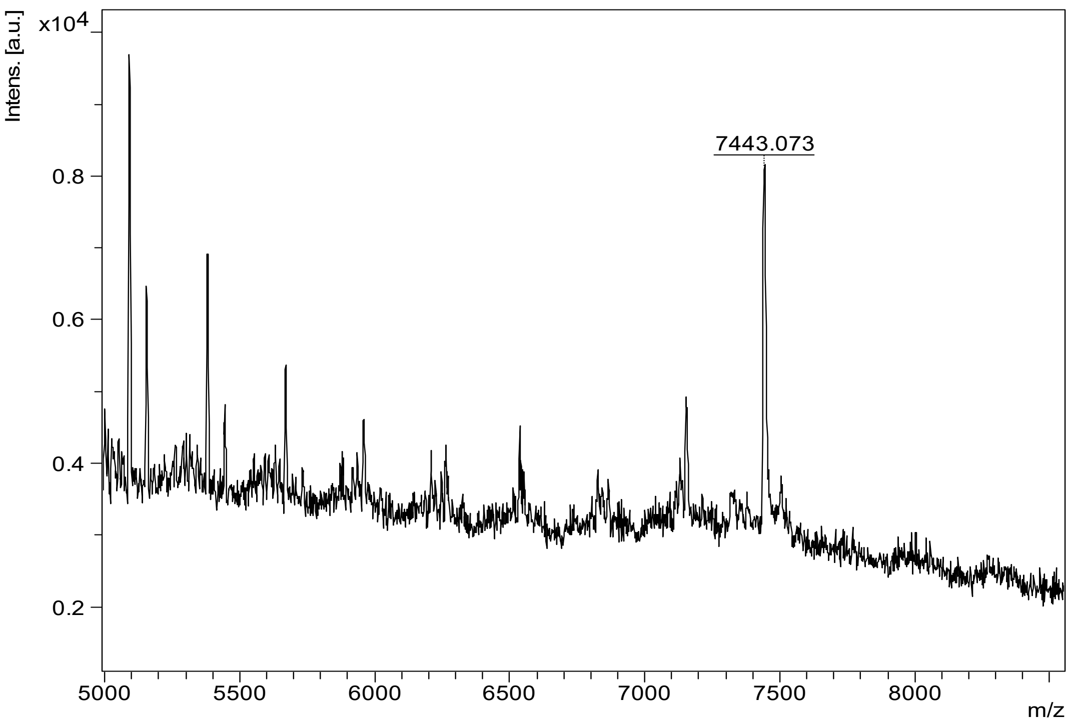

hTERT-26mer-3'T

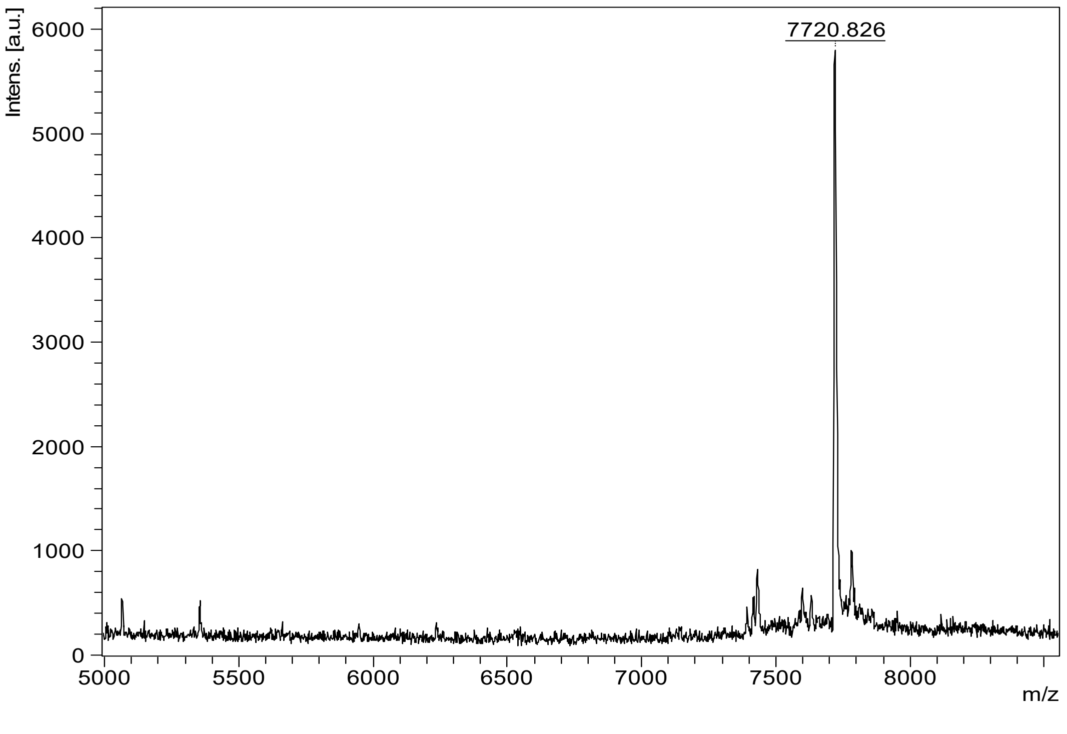

hTERT-19mer-3'C

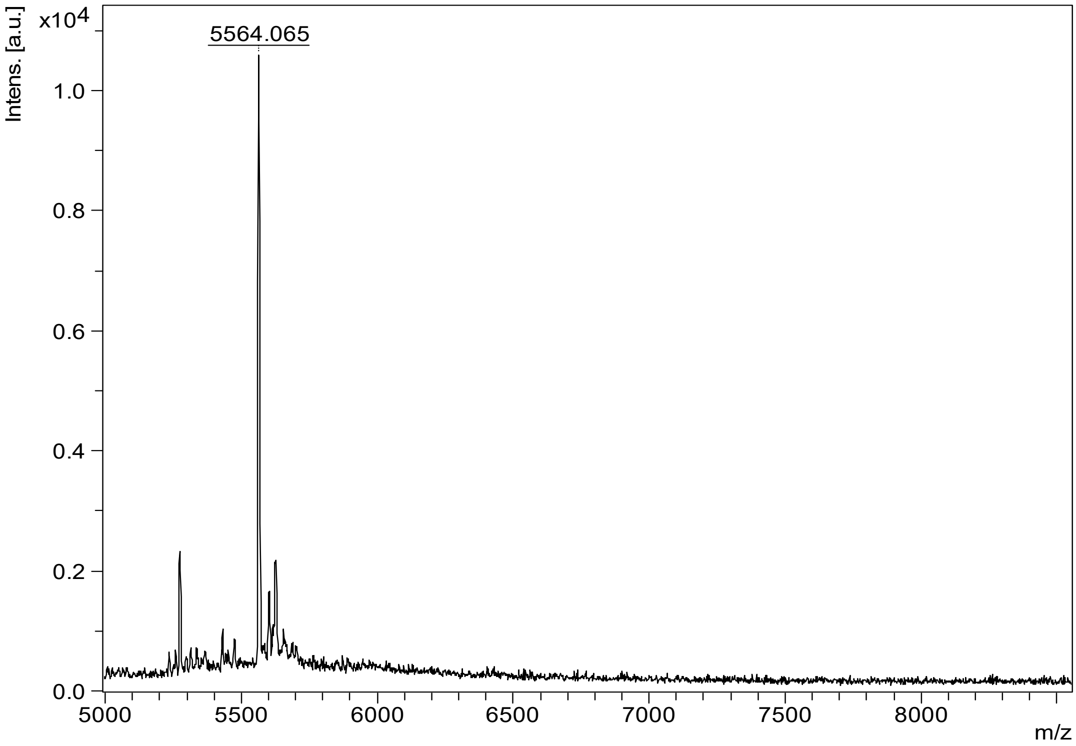
x10^4
Intens. [a.u.]
5564.065
1.0
0.8
0.6
0.4
0.2
0.0
5000
5500
6000
6500
7000
7500
8000
m/z
